# Lower Baseline Serum Triglyceride Levels Are Associated With Higher Decrease in Body Mass Index After Laparoscopy Sleeve Gastrectomy Among Obese Patients

**DOI:** 10.3389/fendo.2021.633856

**Published:** 2021-02-22

**Authors:** Xiu Huang, Guifang Li, Bei Xu, Junyi Zhang, Xingchun Wang, Xiaoyun Cheng, Muthukumaran Jayachandran, Yueye Huang, Shen Qu

**Affiliations:** ^1^ Department of Endocrinology and Metabolism, Shanghai Tenth People’s Hospital, School of Medicine Tongji University, Shanghai, China; ^2^ Shanghai Center of Thyroid Disease, Shanghai, China

**Keywords:** obesity, laparoscopic sleeve gastrectomy, triglyceride, body mass index, waist circumference

## Abstract

**Aims:**

To investigate the predictive value of baseline serum triglyceride (TG) levels for improvements of metabolism after laparoscopic sleeve gastrectomy (LSG).

**Methods:**

112 obese patients [body mass index (BMI) ≥ 35 kg/m^2^] underwent LSG and with complete information of anthropometric and metabolic parameters were divided into normal TG group (group A) and high TG group (group B), while group A had TG levels ≤ 1.7 mmol/L, and group B had TG levels > 1.7 mmol/L. The post-operative changes (Δ) in metabolic parameters between the two groups were compared.

**Results:**

In the whole cohort, the metabolic parameters were significantly improved at 6 months after LSG. BMI and waist circumference (WC) decreased significantly in the two groups. The ΔBMI among group A and group B were 11.42±3.23 vs 9.13±2.77 kg/m^2^ (p<0.001), respectively. ΔBMI was positively correlated with ΔWC (r=0.696, p<0.001), Δfasting insulin level (r=0.440, p=0.002), Δfasting serum C peptide level (r=0.453, p=0.002), and Δhomeostasis model assessment insulin resistance index (r=0.418, p=0.004) in group A. Compared with group B, group A had a significantly higher odds ratio (OR) of 2.83 (95% confidence interval [CI]1.25–6.38, p=0.012)and 2.73 (95% CI 1.11–6.72, p=0.029) for ΔBMI and ΔWC after adjustment for age and gender, respectively.

**Conclusions:**

Obese patients with baseline TG levels under 1.7 mmol/L had greater loss of weight at six months follow-up later LSG. This finding suggests that baseline TG level may have a predictive value for weight loss, at least in the short-term follow-up.

## Introduction

Obesity is a major public health concern globally (1). Based on adipose tissue distribution in the body (upper body and lower body), obesity can be divided into abdominal obesity and gluteofemoral obesity (2). Abdominal obesity is one of the symptoms of metabolic syndrome (MS), and is also a risk factor for cardiovascular disease (CVD) and diabetes mellitus (3, 4). Healthcare professionals have employed several treatment methods to improve weight loss management. Recently, metabolic surgery has become an internationally recognized method for long-term and effective weight control and for improvement in metabolic disorders (5). Laparoscopic sleeve gastrectomy (LSG) is one of the most commonly used methods and has been employed by clinicians globally for many years (6–9). Triglyceride (TG) is an important clinical feature of MS and associated with metabolic abnormalities in nonalcoholic fatty liver disease and abdominal obesity (10, 11). TG is also an independent risk factor for CVD (10). In an analysis of a 23-year cohort study, the first CVD events occurred in half of the patients in the high TG group, and the incidence of having first CVD events was approximately two-fold higher in the high TG group than in the normal TG group (12). However, the prognostic value of TG in obese patients treated with LSG remains unknown.

We aimed to investigate the potential predictive value of baseline TG level on several metabolic outcomes after LSG, and a retrospective analysis of a cohort of obese patients with different TG levels was performed.

## Materials and Methods

### Subjects

In this retrospective study, we recruited obese patients who underwent standard LSG at the Department of Gastrointestinal Surgery in Shanghai Tenth People’s Hospital affiliated with Tongji University from May 2015 to July 2019, and the selection criteria were as follows:

(1) BMI ≥ 35 kg/m^2^;

(2) Good liver and kidney function;

(3) Good cardiopulmonary function;

(4) The follow-up can be completed on time. That is, regular follow-up can be carried out in 1 month, 3 months, and 6 months after surgery;

(5) The TG values were measured before and after surgery.

Patients with a history of any malignant tumor, genetic disease, hypogonadism, renal dysfunction, severe liver dysfunction, preexisting heart disease, and inability to understand and observe the study protocol were excluded. Written informed consent was obtained from each participant before enrolment, and the study protocol was approved by the hospital ethics committee (Clinical Registration Number: ChiCTR-OCS-12002381).

### Anthropometric Assessment and Laboratory Analyses

The height (H), body weight (BW), neck circumference (NC), waist circumference (WC), hip circumference (HC), and BMI were measured at time 0 (pre-operative) and time 6 (6 months after LSG) by trained physicians. Morning venous blood was drawn from the study participants after a 12-h overnight fast, and general biochemical data, including glucose levels, lipid profiles, and liver function markers, were measured at the same period (time 0 and time 6). The levels of FPG, 2-hour plasma glucose (2hPG), fasting insulin (FINS), 2-hour insulin, fasting serum C peptide (FCP), 2-hour serum C peptide, hemoglobin Alc (HbA1c), total cholesterol (TC), TG, HDL-C, low-density lipoprotein cholesterol (LDL-C), and free fatty acid (FFA) were measured using the electrochemiluminescence immunoassay method, as well as the levels of thyroid-stimulating hormone (TSH), free thyroxine, free triiodothyronine (FT3), interleukin 6 (IL-6), interleukin 8 (IL-8), and C-reactive protein (CRP). The homeostasis model assessment insulin resistance index (HOMA-IR) was calculated as following: FPG (mmol/L) ×FINS (mU/L)/22.5.

Patients were divided into two groups based on their serum levels of TG at baseline; group A with TG levels ≤ 1.7 mmol/L, and group B with TG levels > 1.7 mmol/L.

### Statistical Analysis

All continuous data were presented as means ± standard deviation (SD). Independent Student’s t-tests were used to compare all parameters between groups, and paired sample t-tests were used to analyze differences between continuous variables before and after surgery. Pearson’s correlation analysis was used to evaluate the correlation between BMI and metabolic parameters. A binary logistic regression was performed to analyze the predictive indicators of the weight loss effect. All statistical analyses were performed using SPSS 22.0 software (SPSS, Inc., New York, NY, USA), and p-values <0.05 were considered statistically significant.

## Results

### Comparisons Between Metabolic Parameters in Patients With Different TG Levels at Baseline and 6 Months After LSG

A total of 112 (53 males, and 59 females) obese patients with a mean age of 31.15 ± 11.07 years were investigated in the present study. 61 (27 males, and 34 females) patients with a mean age of 27.97 ± 9.60 years were in group A, while 51 (26 males, and 25 females) patients with a mean age of 34.96 ± 11.59 years were in group B.

Great improvements in TG and HDL levels of the two groups were obtained in 6 months after LSG (p<0.01). Similarly, the BMI, NC, WC, WHR, and levels of FPG, FINS, FCP, HbA1c, and HomA-IR were also significantly improved after LSG in both groups (p<0.01).

The pre-operative TC levels in group B was higher than those in group A (4.81±1.06 vs 4.29±0.96 mmol/L, p=0.009); however, there was no difference between the two groups after surgery (4.49±0.90 vs 4.37±0.76 mmol/L, p=0.465). The HDL levels in group B was lower than those in group A before surgery (0.93±0.18 vs 1.04±0.21 mmol/L, p=0.005), and there was no difference after surgery (1.14±0.25 vs 1.20±0.32 mmol/L, p=0.253). Furthermore, pre-operative levels of LDL and FFA did not significantly differ between the two groups, while FFA decreased in group B 6 months after surgery (p<0.05, **Table 1**).

**Table 1:** Baseline clinical characteristics and metabolic parameters at 6 months after laparoscopic sleeve gastrectomy (LSG) in patients with different triglyceride (TG) Levels.

	Baseline			6 months		
	Group A (n = 61)	Group B (n = 51)	p1	Group A (n = 61)	Group B (n = 51)	p2
Age (years)	27.97 ± 9.60	34.96 ± 11.59	0.001	/	/	/
BMI (kg/m^2^)	41.79 ± 6.75	38.49 ± 5.32	0.005	30.37 ± 5.32**	29.35 ± 4.93**	0.298
NC (cm)	43.89 ± 5.06	43.82 ± 4.97	0.941	38.93 ± 4.06**	38.98 ± 3.79**	0.954
WC (cm)	126.71 ± 11.88	120.10 ± 12.27	0.006	100.22 ± 14.23**	99.20 ± 12.13**	0.708
HC (cm)	123.47 ± 15.53	119.82 ± 12.80	0.186	108.46 ± 12.12**	105.56 ± 10.34**	0.208
WHR (%)	0.97 ± 0.07	1.00 ± 0.08	0.058	0.92 ± 0.06**	0.94 ± 0.05**	0.167
SBP (mmHg)	135.82 ± 14.60	135.61 ± 17.51	0.945	123.08 ± 12.48**	125.66 ± 16.19**	0.358
DBP (mmHg)	84.03 ± 12.13	82.94 ± 11.37	0.625	75.00 ± 11.90**	76.80 ± 10.94**	0.409
FPG (mmol/L)	5.63 ± 1.27	7.11 ± 2.75	0.001	4.48 ± 0.41**	4.74 ± 1.09**	0.115
2hPG (mmol/L)	8.68 ± 3.33	11.94 ± 5.05	<0.001	4.07 ± 1.24**	4.99 ± 2.90**	0.042
FINS (mU/L)	33.30 ± 22.23	37.08 ± 30.08	0.461	10.40 ± 6.39**	10.55 ± 5.42**	0.898
2hINS (mU/L)	177.67 ± 137.85	158.64 ± 125.51	0.453	27.87± 33.55**	54.13 ± 68.12**	0.017
FCP (ng/ml)	4.62 ± 2.16	5.33 ± 2.85	0.149	2.57 ± 1.67**	2.66 ± 0.76**	0.684
2hCP (ng/ml)	13.19 ± 6.40	13.67 ± 8.44	0.743	5.9 ± 3.97**	8.94 ± 5.09**	0.001
HomA-IR	8.66 ± 6.94	11.71 ± 10.77	0.088	2.08 ± 1.33**	2.30 ± 1.54**	0.436
HbA1c (%)	6.20 ± 1.14	6.92 ± 2.28	0.047	5.26 ± 0.34**	5.37 ± 0.60**	0.259
TC (mmol/L)	4.29 ± 0.96	4.81 ± 1.06	0.009	4.37 ± 0.76	4.49 ± 0.90	0.465
TG (mmol/L)	1.23 ± 0.29	3.26 ± 2.88	<0.001	0.84 ± 0.25**	1.43 ± 1.22**	0.001
LDL-C (mmol/L)	2.74 ± 0.81	2.78 ± 0.97	0.846	2.84 ± 1.16	2.80 ± 0.81	0.836
HDL-C (mmol/L)	1.04 ± 0.21	0.93 ± 0.18	0.005	1.20 ± 0.32**	1.14 ± 0.25**	0.253
FFA (mmol/L)	0.51 ± 0.19	0.54 ± 0.18	0.414	0.59 ± 0.39	0.48 ± 0.19*	0.044
CRP (mg/L)	7.41 ± 5.45	5.78 ± 3.52	0.061	3.95 ± 2.11**	3.59 ± 1.61**	0.339
IL-6 (pg/ml)	8.68 ± 18.11	19.21 ± 51.83	0.190	4.43 ± 3.65	8.60 ± 19.20	0.153
IL-8 (pg/ml)	261.89 ± 608.00	297.64 ± 568.81	0.759	176.26 ± 341.32	158.32 ± 269.26	0.765
FT3 (pmol/L)	5.14 ± 0.52	4.82 ± 0.78	0.016	4.59 ± 0.54**	4.57 ± 0.62*	0.873
FT4 (pmol/L)	16.26 ± 2.63	16.67 ± 2.67	0.421	15.83 ± 2.59	15.53 ± 2.11**	0.501
TSH (pmol/L)	3.08 ± 1.55	3.91 ± 7.23	0.425	2.31 ± 1.93**	2.47 ± 1.73	0.646

Data are expressed as means ± SD. The independent sample T-test was used to compare the metabolic indicators between group A and group B. p＜0.05 was considered as statistically significant; p1 refers to the comparison between the two groups at baseline, and p2 refers to the comparison between the two groups 6 months after LSG surgery.

Compared with baseline values using a paired T-test, *P＜0.05, **P＜0.01.

LSG, laparoscopic sleeve gastrectomy; BMI, body mass index; NC, neck circumference; WC, waist circumference; HC, hip circumference; WHR, waist/hip ratio; SBP, systolic blood pressure; DBP, diastolic blood pressure; FPG, fasting plasma glucose; 2hPG, 2 hours plasma glucose; FINS, fasting serum insulin; 2hINS, 2 hours serum insulin; FCP, fasting serum C peptide; 2hCP, 2 hours serum C peptide; HOMA-IR, homeostasis model assessment of insulin resistance; HbA1c, glycosylated hemoglobin; TC, total cholesterol; TG, triglyceride; HDL-C, high-density lipoprotein cholesterol; LDL-C, low-density lipoprotein cholesterol; FFA, free fatty acid; IL-6, interleukin 6; IL-8, interleukin 8; CRP, C-reactive protein; FT3, free triiodothyronine; FT4, free thyroxine; TSH, thyroid-stimulating hormone.

As **Table 1** showed, group A had lower preoperative levels of FPG (p<0.01), 2hPG (p<0.01), and HbA1c (p<0.05) than group B. Significant improvements in blood glucose and islet function in both groups were observed after LSG (p<0.01).

### Comparison of the Changes (Δ) in Metabolic Parameters Between Group A and Group B After LSG

Six months after LSG, we found that ΔBMI, ΔWC, and ΔHC in group A were significantly improved than group B (**Table 2**). However, the improvements in TC, TG, FFA, FPG, and 2hPG levels were greater in group B than in group A (p<0.05, **Table 2**).

**Table 2:** Comparison of the improvements in metabolic parameters in patients with different triglyceride (TG) Levels.

	group A	group B	p
△BMI (kg/m^2^)	11.42 ± 3.23	9.13 ± 2.77	<0.001
△NC (cm)	5.31 ± 2.39	4.89 ± 3.14	0.482
△WC (cm)	24.62 ± 8.92	20.26 ± 9.64	0.025
△HC (cm)	19.15 ± 6.13	14.52 ± 8.63	0.004
△WHR (%)	0.05 ± 0.06	0.06 ± 0.06	0.735
△FPG (mmol/L)	1.15 ± 1.28	2.37 ± 2.79	0.005
△2hPG (mmol/L)	4.64 ± 3.24	7.13 ± 4.76	0.003
△FINS (mU/L)	22.69 ± 20.10	26.56 ± 28.19	0.419
△2hINS (mU/L)	143.59 ± 135.14	103.97 ± 129.40	0.129
△FCP (ng/ml)	2.04 ± 2.49	2.67 ± 2.75	0.213
△2hCP (ng/ml)	7.23 ± 7.26	4.71 ± 9.02	0.124
△HomA-IR	6.54 ± 6.53	9.41 ± 10.61	0.101
△HbA1c (%)	0.94 ± 1.05	1.57 ± 2.21	0.075
△TC (mmol/L)	-0.08 ± 0.83	0.32 ± 1.14	0.040
△TG (mmol/L)	0.39 ± 0.30	1.83 ± 3.16	0.002
△LDL-C (mmol/L)	-0.09 ± 1.12	-0.01 ± 0.97	0.661
△HDL-C (mmol/L)	-0.09 ± 0.70	-0.22 ± 0.21	0.168
△FFA (mmol/L)	-0.08 ± 0.43	0.07 ± 0.18	0.017
△CRP (mg/L)	3.54 ± 4.62	1.83 ± 3.11	0.028
△IL-6 (pg/ml)	4.13 ± 19.02	12.74 ± 58.62	0.360
△IL-8 (pg/ml)	90.27 ± 747.35	94.11 ± 417.03	0.975
△FT3 (pmol/L)	0.56 ± 0.58	0.27 ± 0.75	0.028
△FT4 (pmol/L)	0.43 ± 2.93	1.22 ± 2.50	0.132
△TSH (pmol/L)	0.03 ± 6.10	1.50 ± 6.88	0.248

△was calculated as changes in metabolic variables (6-month baseline). The independent sample T-test was used to compare the metabolic indicators between group A and group B. p values < 0.05 were considered as statistically significant.

BMI, body mass index; NC, neck circumference; WC, waist circumference; HC, hip circumference; WHR, waist/hip ratio; FPG, fasting plasma glucose; 2hPG, 2 hours plasma glucose; FINS, fasting serum insulin; 2hINS, 2 hours serum insulin; FCP, fasting serum C peptide; 2hCP, 2 hours serum C peptide; HOMA-IR, homeostasis model assessment of insulin resistance; HbA1c, glycosylated hemoglobin; TC, total cholesterol; TG, triglyceride; HDL-C, high-density lipoprotein cholesterol; LDL-C, low-density lipoprotein cholesterol; FFA, free fatty acid; IL-6, interleukin 6; IL-8. interleukin 8; CRP, C-reactive protein; FT3, free triiodothyronine; FT4, free thyroxine; TSH, thyroid-stimulating hormone.

### Correlations Between ΔTG, as well as ΔBMI and Metabolic Parameters’ Changes in Patients After LSG

The ΔTG level was positively correlated with ΔFPG (r=0.410, p=0.001), Δ2hPG (r=0.349, p=0.008), and ΔHbA1c levels (r=0.267, p=0.045) in group A (**Table 3**). ΔTG level was positively correlated with the ΔFPG (r=0.562, p<0.001), Δ2hPG (r=0.435, p=0.003), ΔHbA1c (r=0.341, p=0.020), and ΔFFA levels (r=0.412, p=0.004), while the ΔTG was negatively correlated with ΔHDL-C (r= -0.307, p=0.034) and ΔLDL-C levels (r= - 0.294, p=0.043) in group B. Similarly, ΔBMI was positively correlated with ΔFPG (r=0.410, p=0.016), ΔFINS (r=0.423, p=0.013), ΔFCP (r=0.572, p<0.001) levels, and ΔHOMA-IR (r=0.540, p=0.001) in group B (**Table 4**).

**Table 3:** Correlations between ΔTG and metabolic parameters’ changes in patients with different triglyceride (TG) Levels.

	group A		group B	
	r	p	r	p
△BMI (kg/m^2^)	0.020	0.879	0.240	0.097
△FPG (mmol/L)	0.410	0.001	0.562	<0.001
△2hPG (mmol/L)	0.349	0.008	0.435	0.003
△FINS (mU/L)	-0.050	0.714	-0.008	0.956
△2hINS (mU/L)	-0.039	0.776	-0.185	0.224
△FCP (ng/ml)	-0.085	0.524	0.108	0.465
△2hCP (ng/ml)	-0.012	0.928	-0.037	0.810
△HomA-IR	0.069	0.610	0.218	0.137
△HbA1c (%)	0.267	0.045	0.341	0.020
△TC (mmol/L)	0.187	0.156	0.182	0.210
△HDL-C (mmol/L)	-0.186	0.159	-0.307	0.034
△LDL-C (mmol/L)	-0.174	0.188	-0.294	0.043
△FFA (mmol/L)	-0.086	0.520	0.412	0.004

△was calculated as changes in metabolic variables (6-month baseline). p values < 0.05 were accepted as statistically significant. All data were adjusted for age and gender.

**Table 4:** Correlations between ΔBMI and metabolic parameters’ changes in patients with different triglyceride (TG) Levels.

	group A		group B	
	r	p	r	p
△WC(cm)	0.696	<0.001	0.689	<0.001
△FPG(mmol/L)	0.186	0.221	0.410	0.016
△2hPG(mmol/L)	0.086	0.576	0.333	0.055
△FINS(mU/L)	0.440	0.002	0.423	0.013
△2hINS(mU/L)	0.208	0.171	0.082	0.646
△FCP(mU/L)	0.453	0.002	0.572	<0.001
△2hCP(mU/L)	0.070	0.646	0.138	0.437
△HOMA-IR	0.418	0.004	0.540	0.001
△HbA1c (%)	0.093	0.544	0.263	0.132
△TC(mmol/L)	-0.166	0.276	0.297	0.088
△TG(mmol/L)	0.074	0.631	0.429	0.011
△LDL-C(mmol/L)	-0.119	0.435	0.106	0.550
△HDL-C(mmol/L)	-0.120	0.431	-0.258	0.141
△FFA(mmol/L)	-0.033	0.830	0.264	0.132

△was calculated as changes in metabolic variables (6-month baseline). p values < 0.05 were accepted as statistically significant. All data were adjusted for age and gender.

BMI, body mass index; WC, waist circumference; FPG, fasting plasma glucose; 2hPG, 2 hours plasma glucose; FINS, fasting serum insulin; 2hINS, 2 hours serum insulin; FCP, fasting serum C peptide; 2hCP, 2 hours serum C peptide; HOMA-IR, homeostasis model assessment of insulin resistance; HbA1c, glycosylated hemoglobin; TC, total cholesterol; TG, triglyceride; HDL-C, high-density lipoprotein cholesterol; LDL-C, low-density lipoprotein cholesterol; FFA, free fatty acid.

### The Relationship Between TG Levels and Effectiveness of LSG by Binary Logistic Regression Analysis

The median values (13) for the ΔBW, ΔBMI, and ΔWC 6 months after LSG were used as the limits; these were 28.70 kg, 10.13 kg/m^2^, and 22.00 cm, respectively. Compared with patients in group B, the odds ratios (ORs) for the ΔBW, ΔBMI, and ΔWC were 2.23(95% confidence interval [CI] 1.04–4.77, p=0.038), 2.78 (95%CI 1.29–6.00, p=0.009), and 2.09(95%CI 0.92–4.76, p=0.078) for group A patients. After adjustment for age and gender, the ORs for ΔBMI, ΔWC, and ΔBW were 2.83(95% CI 1.25–6.38, p=0.012), 2.73(95% CI 1.11–6.72, p=0.029), and 2.20(95% CI 0.96–5.02, p=0.061), respectively (**Table 5**).

**Table 5:** Binary logistic regression analysis for weight loss effect.

Group		n/N (%)	Unadjusted	p	Adjusted^a^	p
			OR (95%CI)		OR (95%CI)	
△body weight						
	group B	20/51(39.22)	Ref.		Ref.	
	group A	36/61(59.02)	2.23(1.04,4.77)	0.038	2.20(0.96,5.02)	0.061
△BMI						
	group B	19/51(37.25)	Ref.		Ref.	
	group A	38/61(62.30)	2.78(1.29,6.00)	0.009	2.83(1.25,6.38)	0.012
△WC						
	group B	17/44(38.64)	Ref.		Ref.	
	group A	29/51(56.86)	2.09(0.92,4.76)	0.078	2.73(1.11,6.72)	0.029

^a^Adjusted age and gender.

Based on the median of △body weight, △BMI, and △WC at 6 months after surgery as the limits, they were 28.70 kg, 10.13 kg/m^2^, and 22.00 cm, respectively. Exceeding this limit is considered effective for weight loss.

## Discussion

The TG level is an important indicator of the metabolic status in patients with obesity, and it is a component of the MS diagnostic criteria (14, 15). However, the association between baseline TG levels and LSG in patients with obesity remains unknown. In this study, we investigated the differences in patients’ metabolic parameters as a result of different baseline TG levels after LSG. The present study demonstrated that the levels of the blood glucose and lipids in both group A and B were significantly improved at 6 months after LSG, but it is worth highlighting that patients with a normal TG had greater improvements in BMI and WC.

Weight-loss surgery has been found to regulate BW safely and effectively, and improve metabolic parameters such as blood glucose and lipid levels (16, 17), as was also demonstrated in this study. BMI and WC, blood pressure, blood glucose and, blood lipids levels, insulin resistance, and HbA1c level were significantly improved after LSG. LSG has shown good efficacy and a low rate of complications and therefore, it is widely used worldwide (18–21). While LSG primarily exerts its restrictive effect by reducing stomach volume, and other metabolic and hormonal effects, it also improves serum lipid levels (22). Varlik et al. (23) found that TG and TC levels decreased significantly compared with preoperative levels in the dyslipidemia group, but not in the groups with normal lipid levels. They also found that LDL-C levels were significantly decreased, while HDL-C levels were significantly increased in both groups. Other research by our team has also extensively confirmed the benefits of LSG surgery for obese patients. The results demonstrate that 6 months after LSG surgery, total testosterone level increases and, fat mass decreases in all regions in obese male patients (24); moreover, the increased testosterone level negatively correlated with FINS and HOMA-IR, especially with IL-6 in acanthosis nigricans (AN) patients. Thus, LSG surgery can improve the skin condition of obese patients with AN (25). Similarly, 12 months after LSG, the subclinical hypothyroidism incidence, TSH levels, and inflammatory markers such as IL-6, TNF-α, and CRP also decrease significantly (5). In this study, patients significantly improved BMI, NC, WC, HC, waist-hip rate(WHR), and FPG, 2hPG, FINS, 2hINS, FCP, 2hCP, HbA1c, TG, HDL-C, CRP, and FT3 levels, and HOMA-IR in both groups.

The decrease in WC and BMI in group A is more significant, suggesting that the initial TG level has a certain prognostic value for the weight loss effect after LSG. To explore the effect of TG on weight loss, we performed regression models for the two groups of patients to evaluate the predictive effect of TG on the ΔBW, ΔBMI, and ΔWC. After adjustment for age and gender, the BMI and WC of patients in group A decreased greater than those in group B.

High TG levels are toxic to the body, resulting in poor blood glucose control, as well as disordered lipids and inflammatory factors levels. Studies have shown that high TG in mouse models can cause an increased level of reactive oxygen species (ROS), and increased activity of myeloperoxidase and adenosine deaminase, leading to inflammation (26). In this study, we observed a trend suggesting that group B patients had high levels of serum inflammatory markers, such as IL-6 and IL-8, than those in group A. Moreover, compared with the baseline values, the IL-6 and IL-8 serum levels also decreased. Zhu et al. (25) found that the IL-6 and IL-8 levels also decreased significantly after LSG in obese Chinese men with AN.

Additionally, a study by Pia Lundman et al. (27) showed that in patients with high TG levels, their plasma IL-6 levels were increased, and this was accompanied by a rise in other inflammation-related biochemical markers and the activation of endothelial cells. Similarly, another cross-sectional study also found that MS patients with hypertriglyceridemia had a significant increase in plasma IL-6 levels, and that IL-6 was positively correlated with HOMA-IR (28). We also found that the ΔTG level was positively correlated with the ΔFFA level in the high-TG group. Coincidentally, a study by Limin Wang et al. (29) found that triglyceride-rich lipoprotein can release neutral and oxidized FFA fragments during the degradation process, thereby, activating the NADPH enzyme and inducing the expression of cytochrome 450-mediated ROS products, which can cause endothelial cell inflammation. Interestingly, an *in vitro* experiment found that 17β-estradiol can decrease the level of TGs in adipocytes, but this effect can be attenuated by the large amount of IL-6 produced by LPS. In turn, this indicates that the inflammation state can also affect TG levels, which may be induced by the weakened activity of adipose TG lipase (30). When the body’s pro-inflammatory effect is stronger than the anti-inflammatory effect, the glucose and lipid metabolism in the body will be affected and related diseases will occur.

Obesity is associated with a chronic low-grade inflammatory state (31). Therefore, patients with higher TG levels had a more severe inflammatory state. Therefore, compared with obese patients with normal TG levels, the first step for patients with high TG levels is to improve the body’s inflammatory state and then, lose weight. This might explain our findings in which the BMI and WC among patients in group B did not show the same improvement as those among patients in group A after LSG surgery. However, the mechanism by which LSG could improve the inflammatory condition is yet to be elucidated.

Lowering TG levels can effectively reduce the risk of CVD, especially in the context of insulin resistance or low HDL levels (32, 33). Moreover, a TG reduction of ≥30% will produce a reduction in 1-year medical expenditure (34). Based on our study, baseline TG level may have a predictive value for weight loss in the short-term follow-up. In addition, obese patients with high TG levels can be treated to lower TG before LSG to improve their inflammatory state and increase the benefits of LSG for this kind of patient.

Patients with obesity or MS accompanied by insulin resistance tend to have TG enrichment and low HDL-C level due to decreased lipoprotein lipase activity. HDL can interchange proteins and lipids under the action of cholesterol ester transfer protein (CETP) and phospholipid transfer protein, thus maintaining the balance between proteins and lipids. In the presence of high TG, HDL level will further decrease, and TG will increase due to CETP (35). In fact, in our study, the HDL-C level in group B was lower than that of group A. A prospective cross-sectional study by Meryem Abi-Ayad et al. (36) found that in patients with MS, lower levels of HDL-C are associated with higher levels of VLDL-TG. Additionally, they found that HDL-C levels (<0.35 g/l), VLDL-TG levels (>0.656 g/l) can predict the presence of atherosclerotic plaque. In recent years, emerging studies have shown that the TG:HDL ratio can replace HOMA-IR and become a sensitive biomarker for early prediction of insulin resistance and cardiometabolic risk in obese people (37–39). Studies have highlighted that HDL can inhibit lipid oxidation and rebuild endothelial cells’ function, and has shown anti-inflammatory and anti-apoptotic activities in animal models (40). Another *in vitro* experiment confirmed that under LPS stimulation, HDL exhibits a wide range of anti-inflammatory activity. Its early anti-inflammatory activity is mainly achieved by reducing the level of Toll-like receptor 4, while the late anti-inflammatory activity is induced by reducing signals related to the interferon receptor pathway (41). From the perspective of anti-inflammatory activity, the decrease in HDL-C level may be closely related to the higher inflammatory state in the high TG group when compared to the normal TG group. Indeed, in our study, correlation analysis showed that the decrease in TG was positively correlated with the increase of HDL-C in group B.

## Conclusions

In conclusion, TG levels play an important role in endocrine dysfunction in patients with obesity, and patients with high TG levels demonstrate slower decreases in body weight parameters (i.e., BMI and, WC), but exhibit rapid improvement in blood glucose and lipid levels. Hence, pre-operative serum TG levels can be used as a predictor of short-term weight loss following LSG.

## Data Availability Statement

The original contributions presented in the study are included in the article/supplementary material. Further inquiries can be directed to the corresponding authors.

## Ethics Statement

The studies involving human participants were reviewed and approved by the ethics committee of Shanghai Tenth People’s Hospital, Tongji University. The patients/participants provided their written informed consent to participate in this study.

## Author Contributions

Conception and design: XH, GL, BX, YH, and SQ. Acquisition, statistical analysis, or interpretation of the data: all authors. Drafting of the manuscript: all authors. Revision of the English language: XC and MJ. All authors contributed to the article and approved the submitted version.

## Funding

This work was supported by the National Key R&D Program of China (2018YFC1314100), sponsored by the Shanghai Pujiang Program (2019PJD040, 2018PJD038), the Natural Science Foundation of China (81970677), the Shanghai Committee of Science and Technology, China (18411951803, 17DZ1910603), and the New Exploration of Blood Glucose Management Mode in Patients with Diabetes Mellitus in Chongming Area of Shanghai (CKY2018-19).

## Conflict of Interest

The authors declare that the research was conducted in the absence of any commercial or financial relationships that could be construed as a potential conflict of interest.
